# Dr. Dawn K. Smith, in memoriam

**DOI:** 10.1002/jia2.26051

**Published:** 2023-01-09

**Authors:** Athena P. Kourtis, Robyn Neblett Fanfair, Kenneth H. Mayer, Jonathan Mermin

**Affiliations:** ^1^ Division of HIV Prevention Centers for Disease Control and Prevention Atlanta Georgia USA; ^2^ Beth Israel Deaconess Medical Center Harvard Medical School and the Fenway Institute Fenway Health Boston Massachusetts USA; ^3^ National Center for HIV Viral Hepatitis STD and TB Prevention Centers for Disease Control and Prevention Atlanta Georgia USA

With the passing of Dawn Kristen Smith, MD, MS, MPH, on 31 October 2022, the HIV world lost a champion for HIV prevention. Dr. Smith was an influential, prolific and pioneering researcher, epidemiologist and public health professional. Her distinguished career focused on using scientific research to answer the most important questions in HIV prevention and health equity.

Among her many accomplishments, Dr. Smith was at the forefront of the development and implementation of pre‐exposure prophylaxis (PrEP) for HIV. Her visionary work moved PrEP to become an essential HIV prevention tool. She served as the lead U.S. Centers for Disease Control and Prevention (CDC) investigator of the first PrEP safety study, a clinical trial of PrEP efficacy in Botswana, and developed CDC's PrEP guidelines that ushered in the PrEP paradigm shift. She conducted studies that illuminated the disproportionate impact of HIV on communities that had traditionally been underrepresented in U.S. research studies, including women, children of persons with HIV, persons who inject drugs and persons in Black/African American communities. During her tenure as a CDC scientist, Dr. Smith always focused on eliminating health disparities in HIV with her studies and making HIV treatment and prevention available to persons in disproportionately affected populations.

Dr. Smith was born in Niles, Michigan in 1949. She received her Doctorate of Medicine degree at the University of Massachusetts Medical School.  She completed her residency at the Indian Health Service Hospital in Fort Defiance, Arizona. She earned dual master's degrees in public health and statistics from the University of Michigan where she began her lifelong commitment as an HIV researcher.

**Figure:**
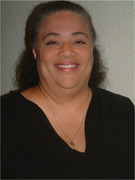
**Dr. Dawn Smith. Photo credit: U.S. Centers for Disease Control and Prevention**.

Dr. Smith came to CDC in 1993 as an Epidemic Intelligence Officer. Some of her many accomplishments include: estimating condom effectiveness; leading the development of the first U.S. guidelines on the use of antiretroviral post‐exposure prophylaxis; identifying gaps in PrEP access and use; increasing understanding of racial, ethnic and economic disparities in access and use of PrEP; increasing understanding of PrEP implementation needs using mathematical modelling; and assessing awareness, knowledge and use of PrEP. She authored more than 100 peer‐reviewed publications and book chapters and presented at numerous conferences. In a highly cited paper, she estimated the number of persons with PrEP indications at the national, state and county levels in the United States [1]. This work provided a denominator for the PrEP coverage indicator used by the CDC and U.S. Department of Health and Human Services to monitor the effectiveness of the U.S. “Ending the HIV Epidemic” initiative and to guide HIV prevention resource allocation decisions. Dr. Smith was the principal investigator of a key study that provided information about real‐world PrEP provision and adherence in community health centres. She also led a study of social networks of Black men who have sex with men with newly diagnosed HIV infection in Alabama, and an analysis of the association between PrEP uptake and HIV incidence in the United States. Up until the end, she was on the front lines of public health, leading the Epidemiology Task Force in CDC's mpox response. Her work estimating PrEP need in the United States informed the development of national goals for mpox vaccine.

Dr. Smith also supported capacity‐building for PrEP implementation in clinical and health department settings. She developed tools to make clinicians and public health workers aware of resources to help pay for PrEP care; clinician training materials; and evidence‐based risk assessment tools for populations disproportionately affected by HIV.

While Dr. Smith's most recent work has focused on PrEP, she was also a champion of women's health and HIV prevention and treatment for women during her entire career. In earlier years, Dr. Smith was a principal investigator of the landmark Human Immunodeficiency Virus Epidemiology Research (HER) Study, a cohort study of HIV infection in U.S. women. She also conducted research on the relationship of injectable hormonal contraception and HIV transmission, the use of vaginal microbicides for HIV prevention and interventions to eliminate mother‐to‐child transmission of HIV.

Dawn was an inspirational, respected and beloved colleague, friend, teacher and mentor, providing insightful guidance and support. She provided a quintessential example of scientific integrity, intellectual rigour and dedication to public health. She used scientific inquiry to examine and bolster arguments, always with the goal of increasing health equity.

Her sudden loss is felt deeply by her family and the broader HIV community. While we grieve the loss of this public health legend and social justice warrior, her legacy lives on through every person who did not get HIV because they had access to, and used, PrEP.

## COMPETING INTERESTS

There are no competing interests from the authors.

## AUTHORS’ CONTRIBUTIONS

APK conceptualized the manuscript and wrote the first draft. RNF reviewed and edited the manuscript. KHM conceptualized the manuscript, reviewed and edited it. JM reviewed and edited the manuscript.

## Data Availability

U.S. CDC Disclaimer: The opinions expressed in this article are those of the authors, and do not necessarily represent the official position of the Centers for Disease Control and Prevention.
